# Comparison of an in‐house hybrid DIR method to NiftyReg on CBCT and CT images for head and neck cancer

**DOI:** 10.1002/acm2.13540

**Published:** 2022-01-27

**Authors:** Chunling Jiang, Yuling Huang, Shenggou Ding, Xiaochang Gong, Xingxing Yuan, Shaobin Wang, Jingao Li, Yun Zhang

**Affiliations:** ^1^ Department of Radiation Oncology Jiangxi Cancer Hospital of Nanchang University Nanchang P. R. China; ^2^ Key Laboratory of Personalized Diagnosis and Treatment of Nasopharyngeal Carcinoma Nanchang Nanchang P. R. China; ^3^ Medical College of Nanchang University Nanchang P. R. China; ^4^ MedMind Technology Co. Ltd. Beijing P. R. China

**Keywords:** cone‐beam CT, deformable image registration, Dice similarity coefficient, free‐form registration, head and neck cancer, similarity metric, viscous fluid registration

## Abstract

An in‐house hybrid deformable image registration (DIR) method, which combines free‐form deformation (FFD) and the viscous fluid registration method, is proposed. Its results on the planning computed tomography (CT) and the day 1 treatment cone‐beam CT (CBCT) image from 68 head and neck cancer patients are compared with the results of NiftyReg, which uses B‐spline FFD alone. Several similarity metrics, the target registration error (TRE) of annotated points, as well as the Dice similarity coefficient (DSC) and Hausdorff distance (HD) of the propagated organs at risk are employed to analyze their registration accuracy. According to quantitative analysis on mutual information, normalized cross‐correlation, and the absolute pixel value differences, the results of the proposed DIR are more similar to the CBCT images than the NiftyReg results. Smaller TRE of the annotated points is observed in the proposed method, and the overall mean TRE for the proposed method and NiftyReg was 2.34 and 2.98 mm, respectively (*p* < 0.001). The mean DSC in the larynx, spinal cord, oral cavity, mandible, and parotid given by the proposed method ranged from 0.78 to 0.91, significantly higher than the NiftyReg results (ranging from 0.77 to 0.90), and the HD was significantly lower compared to NiftyReg. Furthermore, the proposed method did not suffer from unrealistic deformations as the NiftyReg did in the visual evaluation. Meanwhile, the execution time of the proposed method was much higher than NiftyReg (96.98 ± 11.88 s vs. 4.60 ± 0.49 s). In conclusion, the in‐house hybrid method gave better accuracy and more stable performance than NiftyReg.

## 1 INTRODUCTION

In a typical adaptive radiotherapy (ART) process, a cone‐beam computed tomography (CBCT) scan is usually performed to obtain three‐dimensional patient information before treatment.^1^ Since rigid registration is not capable of handling the anatomical variations caused by patient motion, organ filling, tumor growth, or shrinkage with time interval,^2^ it is not suitable for ART. Deformable image registration (DIR) has the ability to depict these local deformations and to estimate the geometric displacement of each image pixel.^3^ It can be used for image fusion,^4^ monitoring the deformation,^5^ and dose mapping.^6^ In the current study of ART, one common way is to deform the planning CT (pCT) image to a CBCT image, and then recalculate the dose based on the deformed image. Therefore, it is necessary to verify the performance of the DIR algorithms.

Currently, there are the geometric, intensity, and deep learning approaches are used for DIR.^7^, ^8^, ^9^ As an intensity approach, free‐form deformation (FFD) assumes that the local deformation is manipulated by a set of control points.^10^ The local deformation in each position is calculated as a weighted sum of the displacements of surrounding control points.^11^ The weightings are related to the distances from the pixels to the surrounding control points.^12^ FFD is efficient because it optimizes the deformation in control points instead of in each pixel, so it has been used in commercial software such as RayStation, MIM Maestro, and Velocity.^13^, ^14^, ^15^, ^16^ RayStation employs an anatomically constrained FFD algorithm,^15^ in which a shape‐based regularization term and a new penalty term are used to keep the deformation reasonable. Velocity employs a B‐Spline FFD algorithm.^16^ Although FFD is widely used, it does not guarantee the preservation of topology.^17^ Different from FFD, the viscous fluid method assumes the image as a viscous fluid whose deformation is governed by the Navier‒Stokes equation.^18^, ^19^ It performs better in topology preservation when the displacements are large. However, it costs more in computation time and great care should be taken in selecting the discretization solver for the partial differential equations.^20^

As an open‐source registration toolkit developed by the Centre of Medical Image Computing at University College London, NiftyReg is an FFD‐based method and has been adopted by many research institutions to do medical image registration.^6^, ^21^, ^22^ It has been confirmed that NiftyReg is able to map identical structures between the CBCT and CT image in most cases.^21^, ^22^, ^23^ However, it is not able to accurately reproduce large or complex anatomical changes, even though it is possible to improve the NiftyReg performance through further optimization of the registration parameters.^21^ There are many parameter options provided in NiftyReg and different combinations of parameter settings will lead to changes in the registration results. Therefore, it is possible to improve the NiftyReg results by optimizing the parameter settings.

To make use of the advantages of different DIR methods, many hybrid methods are proposed.^24^, ^25^, ^26^ For example, the landmark technique was introduced in the FFD methods to solve the different transformations in bony structure and soft tissue.^24^ In this study, an in‐house hybrid method is proposed. It was first tried to use the control points in FFD registration instead of landmark points to accelerate the viscous fluid registration process. The proposed method is applied to transform the pCT images to the CBCT images from 68 patients with head and neck cancer. To verify whether in‐house hybrid methods could improve the registration accuracy, multiple different evaluation methods (points, contours, metrics) are employed to analyze the registration accuracy, and the results of our in‐house hybrid method are compared with those of NiftyReg. It is hoped that this method can be applied to the field of ART in the future.

## 2 MATERIALS AND METHODS

### 2.1 Clinical data

The pCT and the first treatment CBCT data from 68 patients with head and neck cancer were collected for DIR performance evaluation. All the pCT was acquired with a voxel size of 1.27 mm × 1.27 mm × 3 mm by Siemens SOMATOM Definition AS. The treatment CBCT was reconstructed with a resolution of 0.51 mm × 0.51 mm × 1.99 mm by Varian TrueBeam.

### 2.2 Points and OARs delineation

To assess the warp field error near the bone area, five feature points (denoted as P1‒P5) located in different cervical vertebrae are manually labeled in both the CBCT and pCT images of each patient in the same way. P1 and P2 are located on the right and left side of the same vertebra near the oral cavity, P3 and P4 are in the same vertebra near the larynx, and P5 is located in the spinous near the shoulder. Each point is delineated in the axial slice with a small circle. The points with the same index in CBCT and pCT correspond to the same physical position. The pixel position is calculated from the centroid of the small circle. To assess the deformation accuracy in other anatomical structures, five organs at risk (OARs) including the larynx, spinal cord, oral cavity, mandible, and parotid are automatically delineated by MedMind software (MedMind Technology) in both the CBCT and CT images and finally modified by a radiation oncologist.

### 2.3 Data preparation

For each patient, the CBCT data are regarded as the reference image, which would not be warped. Before DIR processing, the input pCT images were processed by the rigid registration. The input pCT for DIR was of the same size and same resolution as the treatment CBCT images. The rigid registration is done by MedMind software.

### 2.4 DIR algorithms

#### 2.4.1 NiftyReg registration

NiftyReg contains tools for global and deformable image registration. The global registration includes rigid registration and affine registration. The deformable registration implementation in NiftyReg uses FFD. Since the deformation is generated from the displacement of control points, its computational consumption can be easily adjusted by setting different numbers of control points. The GPU version of NiftyReg can be obtained from Github.^27^ It is developed in C++ codes and CUDA. It uses multi‐scale registration (three‐level) based on mutual information (MI) optimization. The maximum optimization iteration is set as 30 for each level. The other parameters are set as the default.

#### 2.4.2 In‐house hybrid method

**FIGURE 1:**
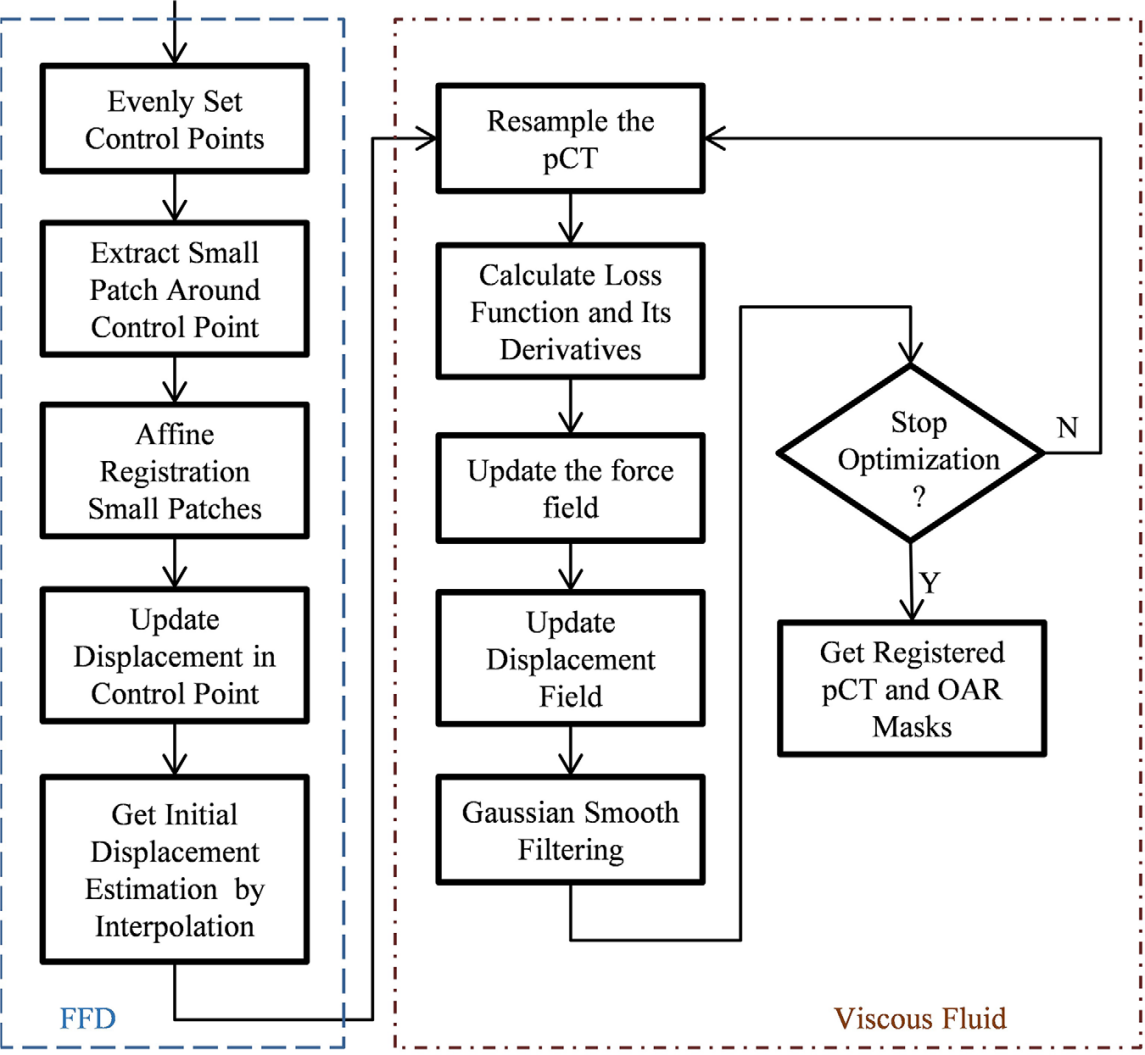
Flowchart of in‐house hybrid method

Due to the usage of interpolation techniques, NiftyReg has a limited ability to depict fine deformation. On the contrary, viscous fluid registration can provide dense displacement for each voxel but it is time consuming. To combine the advantages of the two methods, an in‐house hybrid method is proposed. As shown in Figure 1, it applies FFD with sparsely distributed control points to provide a better initial estimation for viscous fluid flow. Then, the viscous fluid flow is applied to obtain the fine warp field estimation. It also uses the multi‐scale strategy to implement warp field optimization.

Our in‐house hybrid method uses MI as the objective function. The fluid velocity is not calculated from successive over‐relaxation but simplified to a convolution of force field.^19^ The new displacement field is obtained from the current displacement and an increment caused by fluid velocity. Gaussian filtering is further applied to the displacement field to keep the displacement continuous. The pCT image is resampled according to the updated warp field and the optimization steps into the next iteration. The optimization process mentioned above will not stop until the optimization iteration exceeds the maximum optimization iteration or the cost function cannot be further minimized.

### 2.5 Evaluation and statistical tools

After DIR processing, the warping fields define the geometrical displacement between homologous points in the pCT and treatment CBCT images. The warping fields can be used to deform the pCT images to present identical anatomical structures to the CBCT images and to propagate the annotated points and OAR contours defined on pCT to the treatment CBCT.

### 2.6 Intensity‐based metric

Similar to the metrics MI, the normalized cross‐correlation (NCC) and the absolute image value differences (which could directly show the registration errors in bony structure)^28^, ^29^ are indicators for the general registration performance in the whole overlap area. They are used to quantitatively evaluate the registration error in all anatomical structures.

### 2.7 Warp‐based metric

The target registration error (TRE) of the manually annotated points in CBCT and pCT images is used to quantitatively evaluate the accuracy of the warp fields. The Dice similarity coefficient (DSC) between the delineated OAR volume in CBCT and that in warped pCT is a quantitative indicator for the agreement between the deformed pCT OAR volume and the delineated CBCT ones. The DSC is defined as^22^:

(1) $$\mathrm{DSC}\left(A,B\right)=\frac{2\times \left|A\cap B\right|}{\left|A\right|+\left|B\right|}$$

where *A* is the binary mask of an OAR in CBCT and *B* denotes the warped binary mask of the same OAR in pCT. Each OAR is evaluated separately.

The Hausdorff distance (HD) is defined as the maximum of the closest distance between two volumes. The closest distance is computed for each vertex of the two volumes.

(2) $$\mathrm{HD}\left(A,B\right)=\max_{a\in A}\left\{\underset{b\in B}{\min\left\{d\left(a,b\right)\right\}}\right\}$$

The HD is very sensitive to outliers, since the most mismatched point is the sole determining criterion of the distance.

A paired *t*‐test was used for statistical analyses of the two methods, and the *p*‐value ≤ 0.05 was considered statistically significant.

## 3 RESULTS

**FIGURE 2:**
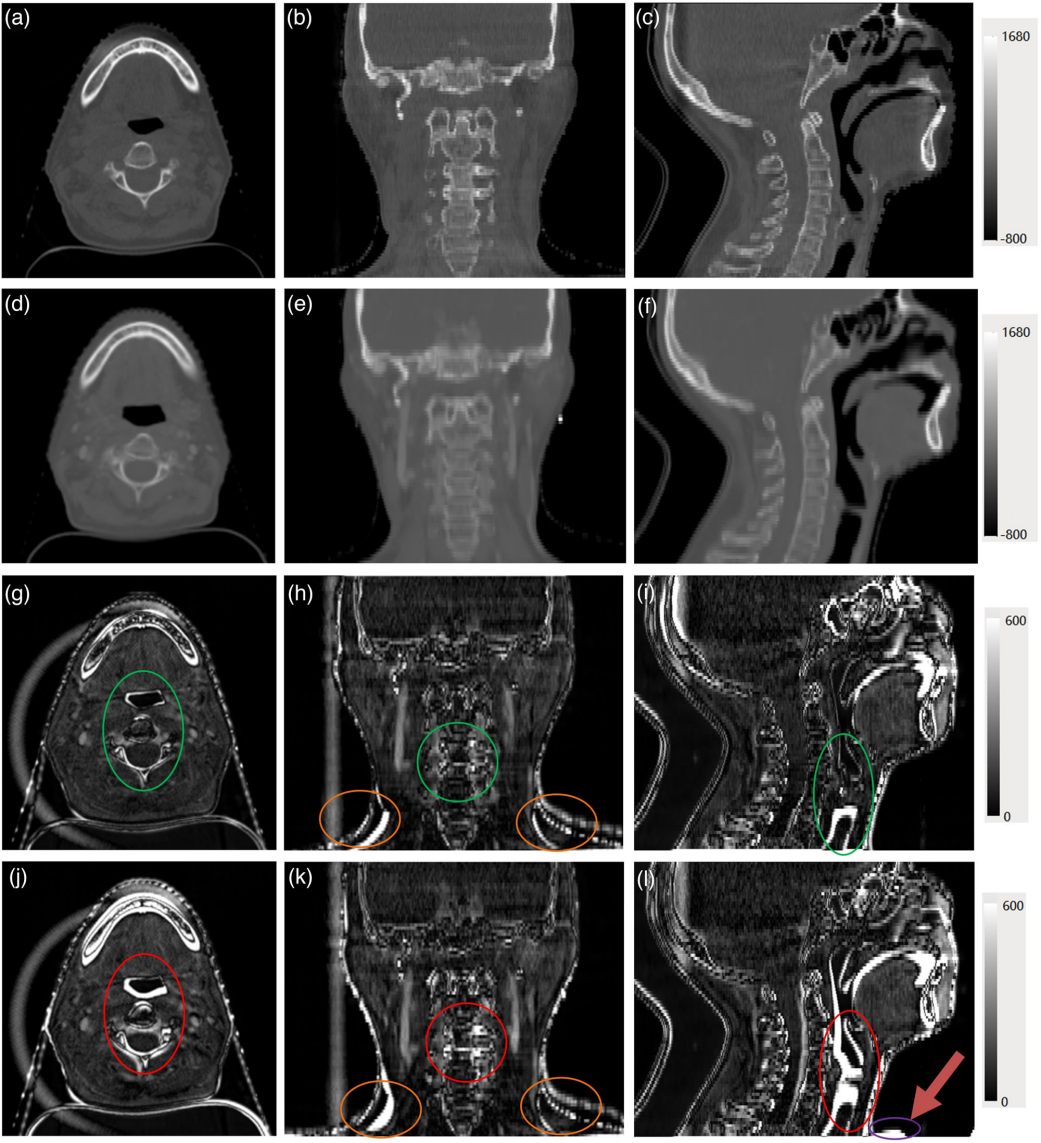
Axial, coronal, and sagittal images between registered computed tomography (CT) and cone‐beam computed tomography (CBCT) image. (a–c) CBCT, (d–f) registered CT, (g–i) difference of in‐house hybrid method, and (j–l) difference of NiftyReg

Figure 2 shows the image value difference between the CBCT images and the warped pCT images given by the two DIR methods. Indicated by the color bar, a larger image difference is shown in a brighter color. It is evident that more bright areas appear in the NiftyReg results, marked by the red circle in Figure 2j–i, whereas the in‐house hybrid method gives darker areas in corresponding green circles. In addition, NiftyReg presents large image discrepancy in the purple circle. In the shoulder area marked by orange circles, large image value differences appear in both methods. As shown in Table 1, the MI and NCC of the proposed DIR are slightly better than those of NiftyReg. The absolute image differences of the proposed method are smaller than the NiftyReg result (*p* < 0.001).

**TABLE 1:** Similarity metrics of registered result

Method	MI	NCC	Absolute difference
In‐house hybrid method	1.18 ± 0.10	0.95 ± 0.02	60.70 ± 11.87
NiftyReg	1.14 ± 0.11	0.95 ± 0.03	67.25 ± 15.68
P	0.000	0.000	0.000

**FIGURE 3:**
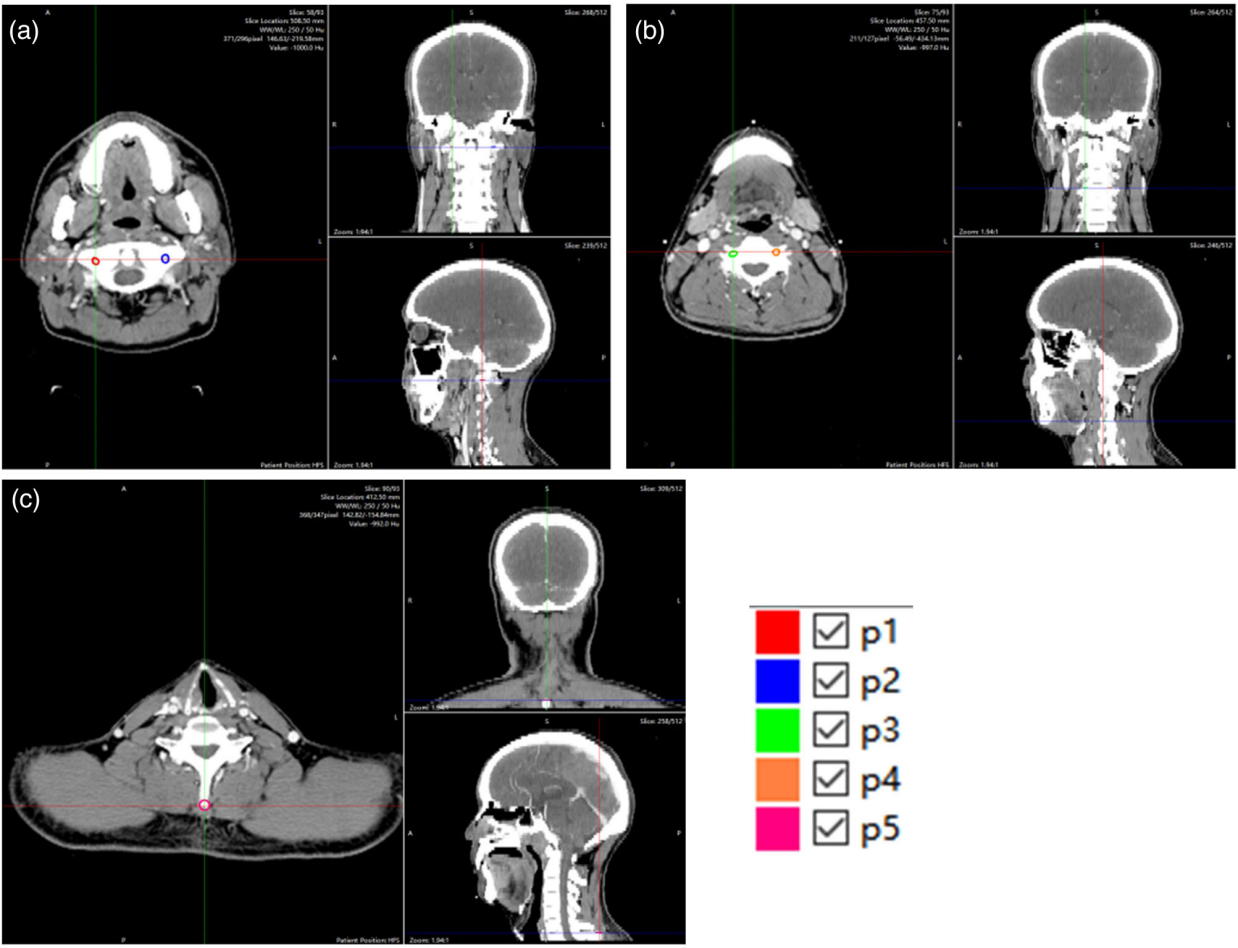
Examples of five annotated points. (a) P1 and P2 are located in the first vertebrae. (b) P3 and P4 are located in the fourth vertebrae. (c) P5 is near the spinous process at the bottom of the image

Five feature points located in different cervical vertebrae are manually labeled in both the CBCT data and CT data (Figure 3). Table 2 lists the mean TRE of the five different positions from P1 to P5. The overall mean TRE in the in‐house hybrid method is lower than the NiftyReg result (2.34 ± 1.02 mm vs. 2.98 ± 1.21 mm). For P1 and P2, NiftyReg gives lower TRE than the in‐house hybrid method, but the difference was not statistically significant.

**TABLE 2:** Mean target registration error (TRE) of annotated points (mm)

**Method**	P1	P2	P3	P4	P5	Overall^*^
In‐house hybrid method	2.13 ± 1.21	2.17 ± 1.68	2.27 ± 1.54	2.16 ± 1.47	2.96 ± 2.24	2.34 ± 1.02
NiftyReg	2.05 ± 1.21	2.04 ± 1.57	3.22 ± 1.84	3.20 ± 1.86	4.41 ± 2.52	2.98 ± 1.21
P	0.258	0.110	0.000	0.000	0.000	0.000

* The average of five points.

**FIGURE 4:**
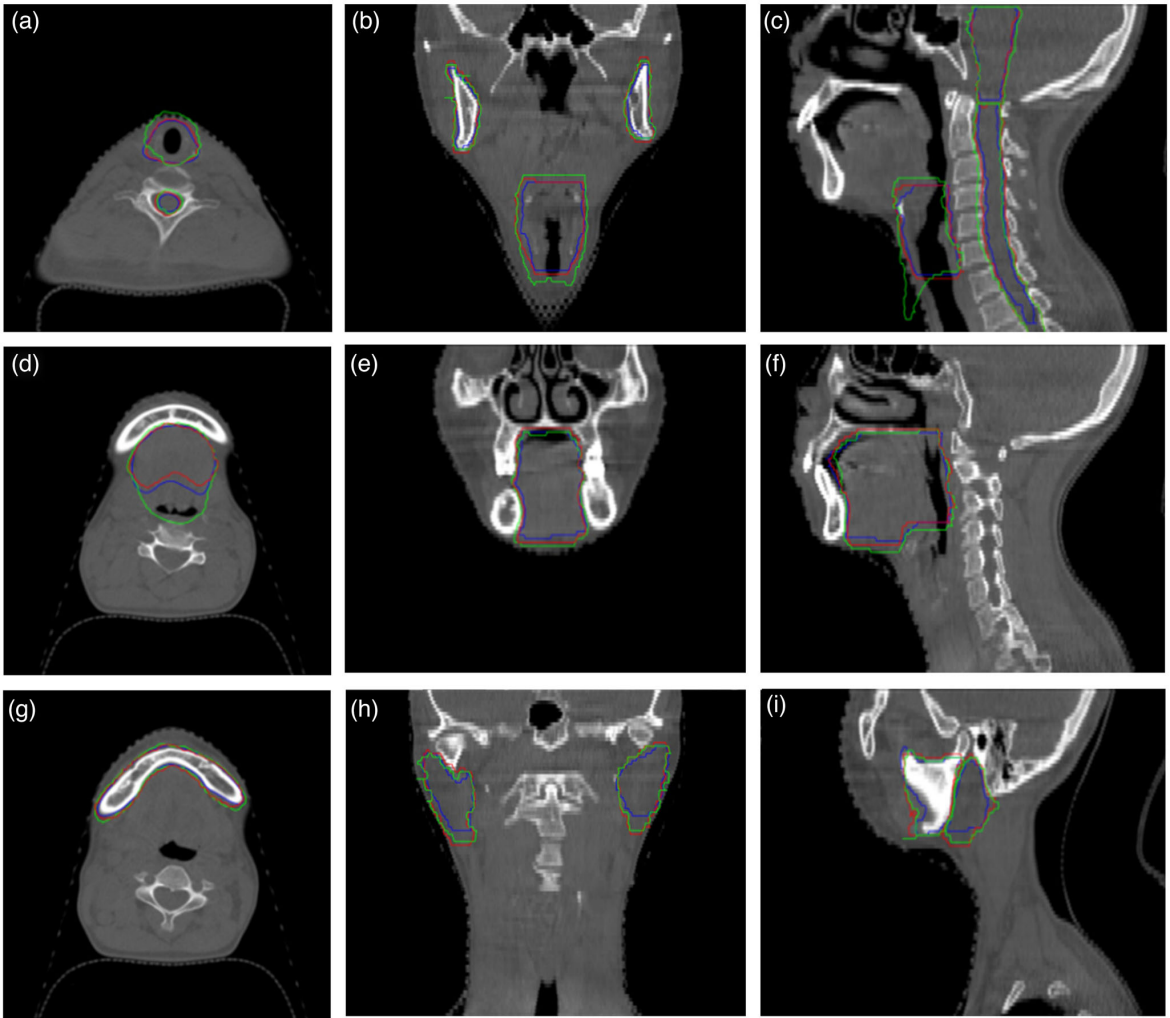
Examples of contour propagation using two deformable image registration (DIR) methods. The blue region is the delineation by cone‐beam computed tomography (CBCT), the red is warped organs at risk (OARs) generated by in‐house hybrid method, and the green is generated by NiftyReg. (a–c) The brain stem, larynx, and spinal cord results; (d–f) the oral cavity; (g–i) mandible, parotid

**FIGURE 5:**
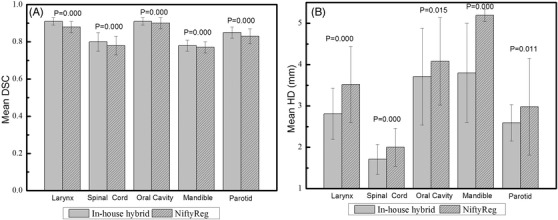
The Dice similarity coefficient (DSC) and Hausdorff distance (HD) of organ at risk (OAR) contours given by in‐house hybrid method and NiftyReg

Figure 4 shows the visual differences between the delineated OARs on the CBCT images and the warped OARs from pCT. In the axial results (a), (d), and (g), the NiftyReg result (shown in the green area) is larger than the blue ground truth. The in‐house hybrid method gives the red result closer to the ground truth. In Figure 4c, unrealistic shape deformation (green part) appears in the NiftyReg result on the bottom of the larynx. For the other OARs such as the oral cavity, mandible, spinal cord, and parotid, the two DIR methods give similar results. Qualitative statistics of DSC and HD in different OARs are presented in Figure 5. As shown in Figure 5, the in‐house hybrid algorithm performs better. The average DSC of the studied OAR results given by the in‐house hybrid method ranged from 0.77 to 0.91, higher than the NiftyReg results (ranging from 0.76 to 0.90), and the HD result of the in‐house hybrid method ranged from 1.71 to 3.80 mm, lower than the NiftyReg results, which ranged from 2.00 to 5.19 (*p* < 0.05).

In addition, it is necessary to compare the execution time of the DIR algorithms. This helps to choose the appropriate DIR algorithm in different RT applications. The average execution time of NiftyReg to register one set of pCT data is about 5 s (4.60 ± 0.49 s), which is much less than the 97 s (96.98 ± 11.88 s) given by the in‐house hybrid method.

## 4 DISCUSSION

In this study, an in‐house hybrid method, combining FFD and a viscous fluid registration method, and NiftyReg were applied to transform pCT to treatment CBCT images. To quantitatively assess their registration accuracy, three intensity‐based similarity metrics were employed to analyze the registration accuracy in the overall overlap. The TRE of annotated points, DSC, and HD of deformed OARs were used to indicate the warp error in different structures. Indicated by the analysis of the different similarity metrics, the in‐house hybrid method gave warped pCT results that were more similar to the CBCT image. From the TREs of the five annotated points, NiftyReg gave more variable performance than the in‐house hybrid method. According to the DSC and HD of different OARs, the in‐house hybrid method performs better than NiftyReg.

As an intensity DIR, FFD uses a set of control points to generate the warp field for each voxel. It gives no consideration to the deformation difference between bony and soft tissue. Therefore, it has a limited ability to handle the deformations of complex structures, such as rigid bony structures surrounded by soft tissues with large deformations. On the contrary, viscous fluid registration provides a dense warp field and it has the potential to depict local warp fields in detail. Enlightened by the hybrid DIR methods,^24^, ^25^, ^26^ the in‐house hybrid method uses FFD registration with sparse control points to provide the initial warp field for the subsequent viscous fluid registration to accelerate the dense warp field optimization process. Due to the neck position changes in the different examinations, FFD is a good choice to correct the large offset in both the head bony structure and the neck bony structure at the same time. That is why FFD shows good results in head and neck applications.^1^, ^14^, ^30^

The image value difference is a direct similarity indicator between the warped pCT and CBCT images. The in‐house hybrid method gives a mean image difference around 60.70, lower than the 67.25 in the NiftyReg results. As shown in the intensity‐based metrics, the in‐house hybrid method gives a higher mean MI and NCC. Seen from both the statistical analysis and the visual comparison, the in‐house hybrid method gives more stable results than NiftyReg. Because no unrealistic deformation of NiftyReg in the HN part was reported in previous studies, the current parameter choices in NiftyReg need to be optimized to reduce the deformation errors.

Similar to the analysis based on landmarks,^31^, ^32^ the TREs of annotated points are used as an indicator for registration error. As shown in Table 2, the TREs of the NiftyReg method results in this study ranged from 2.04 to 4.41 mm, which are similar to the results in other studies.^14^, ^30^ The 4 mm TRE was reported for mono modality image registration,^14^ and the TRE calculated from 14 landmarks was 2.44 mm.^30^ In this study, the TREs of the in‐house hybrid method (2.13 mm in P1, 2.17 mm in P2, 2.27 mm in P3, 2.16 mm in P4, 2.96 mm in P5) are within the voxel diagonal of the pCT voxel diagonal 3.49 mm (1.27 mm × 1.27 mm × 3 mm). According to AAPM TG‐132, the target registration error tolerance should be no more than the maximum volume dimension.^[^ ^13^ ^]^ Therefore, it is possible to use the in‐house hybrid method in medical registration.

Figure 5 shows the mean DSC and HD for different OARs in HN data. The in‐house hybrid method presents a better mean DSC than NiftyReg in the larynx and spinal cord. A lower HD from the in‐house hybrid method is found in most of the analyzed OARs. These DSC statistics are in agreement with results in previous studies.^23^, ^30^, ^33^, ^34^ Bastien showed that the mean DSC for the parotid gland of 15 patients was 0.75.^30^ Raj reported that the B‐spline DIR method gives a mean DSC at 0.74 on HN studies (0.52 for spinal cord, 0.84 for right parotid, 0.79 for left parotid, 0.86 for larynx).^33^ Nobnop applied three different DIR methods to register MV CT images and kV CT images in the HN area,^34^ and the DSC in the right parotid, left parotid, and spinal cord ranged from 0.54 to 0.80. Li tested many different types of DIR methods on CBCT and CT data from HN patients and the mean DSC of the FFD method fell in the range of 0.7 to 0.9.^23^ According to the DSC tolerance for clinical application (from 0.8 to 0.9) in AAPM TG‐132,^13^ both the in‐house hybrid method and NiftyReg can be used for some OARs. The HD of the two methods in this study ranges from 1.76 to 5.16 mm, which is in accordance with the 5 mm reported in the previous study.^13^

The prominent drawback of the in‐house hybrid method is that it consumes too much time, 21 times longer than NiftyReg. This is mainly because the NiftyReg used in this study was a GPU version, and was tested with the extra support of NVIDIA GeForce GTX 1660. Meanwhile, the in‐house hybrid method was developed on a CPU and lacks parallel computing, and the testing environment is Intel i5‐9400F CPU (2.9 GHz) with 16GB RAM. The in‐house hybrid method could be optimized into a CUDA version and the time consumption could potentially be reduced to 30 ms in our estimation. Since the viscous fluid registration process in the in‐house hybrid method needs to optimize the local deformation in each pixel position, its computation takes longer than that of NiftyReg. It should be mentioned that the impact of DIR on dose calculations has not been analyzed in this study. This is the major drawback but it will be studied in further research.

## 5 CONCLUSION

In this study, an in‐house hybrid DIR method and NiftyReg are evaluated to register pCT images to CBCT images in the head and neck region. The in‐house hybrid method gives a better intensity‐based similarity, and the registration error in most of the annotated points (P3, P4, P5), and DSC and HD in most of the tested OAR (larynx, spinal cord, oral cavity, mandible, and parotid) are better than NiftyReg. Furthermore, the in‐house hybrid method presents more stable performance than NiftyReg, and it has the potential to be used in the field of adaptive radiotherapy.

## AUTHOR CONTRIBUTIONS

Chunling Jiang: Data curation, methodology, project administration, and writing–original draft. Yuling Huang: Data curation, formal analysis, and investigation. Shenggou Ding: Investigation and project administration. Xiaochang Gong and Shaobin Wang: Data curation and investigation. Xingxing Yuan: Formal analysis. Jingao Li: Methodology and writing–review. Yun Zhang: Conceptualization, formal analysis, and writing–review and editing.

## References

[1] Li X , Zhang Y , Shi Y , et al. Comprehensive evaluation of ten deformable image registration algorithms for contour propagation between CT and cone‐beam CT images in adaptive head & neck radiotherapy. PLoS One. 2017;12(4):e0175906. 28414799 10.1371/journal.pone.0175906 PMC5393623

[2] Ali I , Alsbou N , Jaskowiak J , Ahmad S . Quantitative evaluation of the performance of different deformable image registration algorithms in helical, axial, and cone‐beam CT images using a mobile phantom. J Appl Clin Med Phys. 2018;19(2):62‐73. 29446235 10.1002/acm2.12246 PMC5849853

[3] Rigaud B , Simon A , Castelli J , et al. Deformable image registration for radiation therapy: principle, methods, applications and evaluation. Acta Oncol. 2019;58(9):1225‐1237. 31155990 10.1080/0284186X.2019.1620331

[4] Nix MG , Prestwich RJD , Speight R . Automated, reference‐free local error assessment of multimodal deformable image registration for radiotherapy in the head and neck. Radiother Oncol. 2017;125(3):478‐484. 29100697 10.1016/j.radonc.2017.10.004

[5] Woerner AJ , Choi M , Harkenrider MM , Roeske JC , Surucu M . Evaluation of deformable image registration‐based contour propagation from planning CT to cone‐beam CT. Technol Cancer Res Treat. 2017;16(6):801‐810. 28699418 10.1177/1533034617697242 PMC5762035

[6] Veiga C , McClelland J , Moinuddin S , et al. Toward adaptive radiotherapy for head and neck patients: feasibility study on using CT‐to‐CBCT deformable registration for "dose of the day" calculations. Med Phys. 2014;41(3):031703. 24593707 10.1118/1.4864240

[7] Crum WR , Hartkens T , Hill DL . Non‐rigid image registration: theory and practice. Br J Radiol. 2004;77:S140‐S153. 15677356 10.1259/bjr/25329214

[8] Fu Y , Lei Y , Wang T , Curran WJ , Liu T , Yang X . Deep learning in medical image registration: a review. Phys Med Biol. 2020;65(20):20TR01. 10.1088/1361-6560/ab843e PMC7759388 32217829

[9] Haskins G , Kruger U , Yan P . Deep learning in medical image registration: a survey. Machine Vision and Applications. 2020;31:1‐2. 10.1007/s00138-020-01060-x

[10] Holden M . A review of geometric transformations for nonrigid body registration. IEEE Trans Med Imaging. 2008 ;27(1):111‐128. 18270067 10.1109/TMI.2007.904691

[11] Rueckert D , Sonoda LI , Hayes C , Hill DL , Leach MO , Hawkes DJ . Nonrigid registration using free‐form deformations: application to breast MR images. IEEE Trans Med Imaging. 1999;18(8):712‐721. 10534053 10.1109/42.796284

[12] Rueckert D , Aljabar P . Non‐rigid registration using free‐form deformations. Springer; 2015.

[13] Motegi K , Tachibana H , Motegi A , Hotta K , Baba H , Akimoto T . Usefulness of hybrid deformable image registration algorithms in prostate radiation therapy. J Appl Clin Med Phys. 2019;20(1):229‐236. 30592137 10.1002/acm2.12515 PMC6333149

[14] Broggi S , Scalco E , Belli ML , et al. A comparative evaluation of 3 different free‐form deformable image registration and contour propagation methods for head and neck MRI: the case of parotid changes during radiotherapy. Technol Cancer Res Treat. 2017;16(3):373‐381. 28168934 10.1177/1533034617691408 PMC5616054

[15] Pukala J , Johnson PB , Shah AP , et al. Benchmarking of five commercial deformable image registration algorithms for head and neck patients. J Appl Clin Med Phys. 2016;17(3):5735. 10.1120/jacmp.v17i3.5735 PMC5690934 27167256

[16] Kadoya N , Nakajima Y , Saito M , et al. Multi‐institutional validation study of commercially available deformable image registration software for thoracic images. Int J Radiat Oncol Biol Phys. 2016;96(2):422‐431. 27475673 10.1016/j.ijrobp.2016.05.012

[17] Tennakoon R , Cao Z , Bab‐Hadiashar A . Nonlinear approaches in three dimensional medical image registration. Springer International Publishing; 2015.

[18] D'Agostino E , Maes F , Vandermeulen D , Suetens P . A viscous fluid model for multimodal non‐rigid image registration using mutual information. Med Image Anal. 2003;7(4):565‐575. 14561559 10.1016/s1361-8415(03)00039-2

[19] Christensen GE , Vannier MW , Chao K , Dempsey JF , Williamson JF . Large‐deformation image registration using fluid landmarks. 2001. 4th IEEE Southwest Symposium on Image Analysis and Interpretation (SSIAI 2000), 2–4 April, Austin, TX.

[20] Alahyane M , Hakim A , Laghrib A , Raghay S . Fluid image registration using a finite volume scheme of the incompressible Navier Stokes equation. Inverse Problems & Imaging. 2018;12:(5):1055–1081. 10.3934/ipi.2018044

[21] Marchant TE , Joshi KD , Moore CJ . Accuracy of radiotherapy dose calculations based on cone‐beam CT: comparison of deformable registration and image correction based methods. Phys Med Biol. 2018;63(6):065003. 29461255 10.1088/1361-6560/aab0f0

[22] Xu Z , Lee CP , Heinrich MP , et al. Evaluation of six registration methods for the human abdomen on clinically acquired CT. IEEE Trans Biomed Eng. 2016;63(8):1563‐1572. 27254856 10.1109/TBME.2016.2574816 PMC4972188

[23] Li X , Zhang YY , Shi YH , Zhou LH , Zhen X . Evaluation of deformable image registration for contour propagation between CT and cone‐beam CT images in adaptive head and neck radiotherapy. Technol Health Care. 2016;24(Suppl 2):S747‐S755. 27259084 10.3233/THC-161204

[24] Paquin D , Levy D , Xing L . Hybrid multiscale landmark and deformable image registration. Math Biosci Eng. 2007;4(4):711‐737. 17924721 10.3934/mbe.2007.4.711

[25] Wörz S , Rohr K . Spline‐Based Hybrid Image Registration using Landmark and Intensity Information based on Matrix‐Valued Non‐radial Basis Functions. International Journal of Computer Vision. 2014;106:(1):76–92. 10.1007/s11263-013-0642-z

[26] Cheng RC , Jian Z , Gang CG . Fast non‐rigid image registration using viscous fluid B‐spline model. J Image Graphics. 2009;14(4):712‐716.

[27] https://github.com/paraq/niftyreg

[28] Zitová B , Flusser J . Image registration methods: a survey. Image and Vision Computing. 2003;21:(11):977–1000. 10.1016/s0262-8856(03)00137-9

[29] Huang Y , Li C , Wang H , et al. A quantitative evaluation of deformable image registration based on MV cone beam CT images: impact of deformation magnitudes and image modalities. Phys Med. 2020;71:82‐87. 32097874 10.1016/j.ejmp.2020.02.016

[30] Rigaud B , Simon A , Castelli J , et al. Evaluation of deformable image registration methods for dose monitoring in head and neck radiotherapy. Biomed Res Int. 2015;2015:726268. 25759821 10.1155/2015/726268 PMC4339705

[31] Sen A , Anderson BM , Cazoulat G , McCulloch MM , Elganainy D , McDonald BA . Accuracy of deformable image registration techniques for alignment of longitudinal cholangiocarcinoma CT images. Med Phys. 2020;47(4):1670‐1679. 31958147 10.1002/mp.14029 PMC7288249

[32] Varadhan R , Karangelis G , Krishnan K , Hui S . A framework for deformable image registration validation in radiotherapy clinical applications. J Appl Clin Med Phys. 2013;14(1):4066. 23318394 10.1120/jacmp.v14i1.4066 PMC3732001

[33] Hardcastle N , Tomé WA , Cannon DM , et al. A multi‐institution evaluation of deformable image registration algorithms for automatic organ delineation in adaptive head and neck radiotherapy. Radiat Oncol. 2012;7:90. 22704464 10.1186/1748-717X-7-90 PMC3405479

[34] Nobnop W , Chitapanarux I , Wanwilairat S , Tharavichitkul E , Lorvidhaya V , Sripan P . Effect of deformation methods on the accuracy of deformable image registration from kilovoltage CT to tomotherapy megavoltage CT. Technol Cancer Res Treat. 2019;18. 10.1177/1533033818821186 PMC6373993 30803375

